# Driving policy dialogue on health technology assessment in Eastern Europe and Central Asia: reporting from an initiative of Health Technology Assessment International

**DOI:** 10.1017/S0266462325000066

**Published:** 2025-02-06

**Authors:** Antonio Migliore, Nicola Vicari, Eva Turk, Rabia Sucu

**Affiliations:** 1 Health Technology Assessment International (HTAi), 1280, 5555 Calgary Trail, Edmonton, Alberta, Canada T6H 5P9; 2 Centre for Digital Health and Social Innovation, University of Applied Sciences St. Poelten, Campusplatz 1, St. Poelten, Austria; 3 Medical Faculty, University of Maribor, 2000 Maribor, Slovenia.

**Keywords:** health technology assessment, Eastern Europe, Central Asia, policy, dialogue

## Abstract

Eastern Europe and Central Asia (EECA) represents a diverse region facing complex healthcare challenges, including resource constraints, fragmented systems, and limited access to evidence-based decision-making tools. Health technology assessment (HTA) offers a critical framework for addressing these issues by informing efficient allocation of healthcare resources. In April 2024, HTA International (HTAi) convened a policy dialogue in Astana, Kazakhstan, bringing together stakeholders from 12 EECA countries and international experts to discuss HTA advancement in the region. The dialogue highlighted systemic barriers, including political instability, capacity shortages, and fragmented data sources while exploring successful HTA implementation models in some countries. Participants emphasized the importance of political commitment, institutional frameworks, and capacity building, alongside fostering stakeholder collaboration. International organizations such as HTAi and WHO were recognized as vital enablers for technical support and knowledge sharing. Key outcomes included actionable recommendations: strengthening political advocacy, developing legal and institutional frameworks, investing in workforce development, and enhancing multistakeholder engagement. The dialogue underscored HTAi’s role in catalyzing regional collaboration, providing platforms for discussion, and offering resources for capacity building. Future initiatives will focus on addressing structural weaknesses, promoting transparency, and embedding HTA into national healthcare systems to ensure equitable and evidence-based decisions. The findings reinforce the potential of HTA to enhance healthcare policy and planning in EECA, fostering resilient systems that better meet population health needs despite ongoing challenges.

## Background

Eastern Europe and Central Asia (EECA) comprises a large group of countries, each with unique historical, political, and economic contexts that influence their healthcare systems. The region is diverse and includes European Union (EU) Member States (such as Poland, Greece, and Slovenia), upper-middle-income countries such as Kazakhstan, Georgia, Serbia, and Ukraine, as well as lower-middle-income countries such as Kyrgyzstan, Uzbekistan, and Tajikistan (1). The transition from centrally planned economies to market-oriented systems in the post-Soviet era has been marked by significant challenges. Many EECA countries face chronic underfunding of healthcare services, leading to significant out-of-pocket payments by patients. This situation is exacerbated by limited public health insurance coverage and inefficiencies in the allocation of healthcare resources. (2). In epidemiological terms, the region is experiencing a dual burden of disease, with high rates of NCDs, such as cardiovascular diseases and diabetes, (3;4) alongside persistent challenges related to infectious diseases, such as tuberculosis and HIV/AIDS (5;6). Access to healthcare services varies widely across the region, with rural populations and marginalized groups often facing significant barriers (7). This disparity is compounded by a shortage of healthcare professionals and outdated healthcare infrastructure in many areas. In addition, the healthcare systems in many EECA countries are further strained by political and economic instability. Frequent changes in government, fluctuating healthcare policies, and a lack of continuity in leadership hamper long-term healthcare planning and the implementation of reforms (8). Despite these challenges, there is a growing recognition of the importance of health technology assessment (HTA) in the EECA region (9) as a critical tool for evidence-based decision-making in healthcare, especially in resource-constrained settings where efficient allocation of resources is paramount (10). HTA provides a structured approach to assess the value of healthcare interventions, helping policymakers prioritize those that offer the greatest benefit relative to their costs (11). To address these challenges and support the advancement of HTA in the EECA region, HTA International (HTAi) has initiated a series of policy dialogues. The first of these was held in Baku, Azerbaijan, in October 2022, and brought together representatives from various EECA countries to discuss the strategic development of HTA (12). The second policy dialogue, held in Astana, Kazakhstan, in April 2024, continued this effort, with participants focusing on practical strategies to advance HTA in their respective countries (13). The meeting was hosted by the Kazakhstan Association of HTA, EBM, and Pharmacoeconomic Research (KazSPOR) concomitantly to their HTA Conference 2024 *“Health Technology Assessment (HTA) as a tool to determine priorities for healthcare development and funding”* which was held on April 19-20, 2024 (program accessible at https://htaconference.kz/en/). The Conference received support of the Ministry of Healthcare of the Republic of Kazakhstan, and the WHO Country Office in Kazakhstan, and this was acknowledged as a strong sign from the institutions to move further toward the implementation and use of HTA in the region.

## Policy dialogue meeting structure

HTAi policy dialogues are intended to underscore the significance of collaboration and co-creation among the stakeholders of various countries, providing a “safe space” that encourages participants to discuss and subsequently collaborate to create sustainable HTA frameworks that can be customized to the specific needs of their respective countries. By fostering a multistakeholder dialogue that includes policymakers, HTA bodies, healthcare providers, and patient representatives, HTAi aims to support the construction of a strong foundation for the future of HTA in the region. HTAi policy dialogues require participants to set aside their personal agendas and virtually disengage from any institutional responsibilities to collaborate on a shared objective: to ensure that HTA is a fully functional and integral component of their healthcare decision-making.

The HTAi Policy Dialogue for EECA in 2024 was a hybrid meeting that saw the participation of 27 individuals from different stakeholder groups, such as governmental institutions, health insurance institutes, and academia, from 12 countries from the region (Azerbaijan, Greece, Hungary, Kazakhstan, Kyrgyzstan, Montenegro, Poland, Serbia, Slovakia, Slovenia, Türkiye, and Ukraine). The event was also attended by experts in HTA from the United States and Italy. The majority of participants attended in person. All participants, regardless of whether they were present in person or virtually, made equal contributions to the conversation.

The meeting commenced with an overview of the objectives and format, which was followed by a presentation from the World Health Organization Office for Europe (WHO EURO) delegates. Subsequently, a roundtable discussion was held, during which all participants were encouraged to provide an update on the status of HTA advancement in their respective countries. This included the sharing of success stories and the identification of current barriers or critical issues. An open discussion took place after the round table and was followed by a final session presenting closing remarks and future possible steps. A meeting report was circulated among the participants, for review and approval, and was the source for the writing of the present article.

## Meeting discussions

### Overview of the WHO Regional Office for Europe activities on HTA and pricing and reimbursement

Delegates from the WHO Regional Office for Europe (WHO Europe) provided an overview of the work of the Access to Medicines and Health Products’ unit to support local governments in activities linked to HTA. The WHO started to assess the value and the status of HTA at the global level in 2015, as a response to the resolution WHA67.23 adopted on 24 May 2014 at the 67th session of the World Health Assembly on Health Intervention and Technology Assessment in support of universal health coverage (14). A global survey on HTA was subsequently launched, and its most recent iteration was conducted in 2020-2021. The results are available through an interactive dashboard that provides detailed results on the key indicators for HTA and health benefit packages (HBP) for all member countries globally. Individual country profiles for both HTA and HBP can be downloaded but also comparative data on the different indicators and countries of interest can be curated and selected through the dashboard. Currently, the survey is being updated to increase the level of detail in terms of data collection and presentation. The revised iteration is expected to be conducted in 2025. The response rate from the WHO European Region was only 66 percent in the last survey giving rise to incomplete information, so work is ongoing to ensure a better response rate for the coming year from the region (15).

The region covered by the WHO Europe is quite large (53 countries) and includes EECA countries. It was reported that several countries have received WHO support in terms of expertise within the framework of bilateral agreements, in particular, Estonia, Latvia, Moldova, Georgia, Greece and Ukraine, while with others the collaboration is going to start or interest has been shown, like in the case of North Macedonia. The WHO Euro Office has continuous dialogue with the former EUnetHTA network, the European Commission (EC) DG SANTE’s HTA unit, the Heads of HTA Agencies Group (HAG), and the Member State Coordination Group on HTA (HTACG) to ensure aligned support to countries.

The WHO Europe especially focused on the pricing and reimbursement in relation to the activities performed in the 2021-2024 timeframe in Central Asia. In the whole subregion, advocacy, policy, and technical meetings have been held on pricing and reimbursement of NCD medicines, and a roadmap for health and well-being was defined in 2023 (16). Customer-tailored support has been provided to three countries, Uzbekistan (e.g., report on availability and prices of essential medicines, price dynamics and consumption patterns analysis, recommendations for strengthening the reference pricing system, internal pricing launch for reimbursable medicines) (17), Tajikistan (report on availability and prices of essential medicines, strengthening pharmaceutical sector, policy dialogue on medicines) (18), and Turkmenistan (webinar on reimbursement, supply chain and procurement).

During the discussion that followed the presentation, three elements that are crucial for HTA implementation activities in the EECA region emerged:

- Political will: It is required to initiate any activity around HTA country support by WHO. Requests for support by WHO for HTA have to come through a formal request from the Ministry of Health, so the central local governments need to work with their ministries to request this. However, the willingness alone is not sufficient, and practical constraints exist that can include, the lack of dialogue among different relevant national institutions or stakeholders. As a neutral convener, WHO can help facilitate the interlinking of national structures and support the strengthening of an HTA system that is more unified and has a better interplay of individual stakeholders with each other. Political instability can also be a major barrier. System building is a time-consuming process, and it is often impossible to complete or observe the impact of the activities that were initiated if political priorities shift.
- Capacity for HTA: As a very large and varied region, the capacity needs can be quite diverse; in some countries, WHO needs to support the development of structures where no activities existed before, while in others, only a tailored approach is needed to strengthen existing HTA capacities, update methods and provide specific training. There is also a key challenge of retaining the trained HTA workforce in EECA countries, once capacity building is conducted.
- Access to data: Due to the variety of sources in the different contexts, availability and access to data are a crucial aspect, particularly for price referencing. Data sources can vary significantly in form, language, presentation, etc.

### Current challenges and priorities to move HTA forward in the region

The participants were invited to reflect and share their thoughts on the challenges they are facing in attempting to make HTA an integral part of their system.

In Kyrgyzstan, the development of HTA was significantly influenced by the numerous political disruptions and changes within the Ministry of Health. A new body was established to deal with HTA, in replacement of the former institution, which was dissolved. The different visions of the different officials in charge, in connection with the changes at the ministerial level, hamper the evolution and growth of HTA which is not institutionalized. Currently, a project funded by the World Bank (2023-2025) aims to provide advice and expertise in the areas of (i) revision of the state health insurance benefit package, including costing using top-down and bottom-up methods (ii) primary care provider payment mechanisms, (iii) pharmaceutical pricing and drug reimbursement/benefit package, and (iv) HTA.

In Azerbaijan, the medical insurance system started to be operational after COVID. In relation to HTA, a formal institutionalization has not been achieved yet. A body or office to host the HTA functions has not yet been identified nor has the necessary staff to run it. It is expected that technical training for staff will be necessary and experiences from other countries as well as support from the WHO could be decisive in moving forward and creating the groundwork for appropriate growth in the field of HTA.

In Turkey, issues in delayed access to innovative therapies, which translates into missed opportunities to improve the healthcare service, are a challenge. HTA is not included in these processes, only budget impact analysis (BIA) is used. There is a department responsible for HTA within the Ministry of Health which started with a grant received in 2012 from the World Bank. Regrettably, funds were not used to create permanent capacity, and functions were largely outsourced. Currently, the biggest challenge is to regain that trust and political will to move forward with HTA. It was highlighted that a technology lifecycle approach would support investment/disinvestment strategies in healthcare.

In Kazakhstan, the journey toward HTA started in 2009 with a project supported by the World Bank. Due to that, Kazakhstan has been acknowledged as a leading country in the region. However, there have been challenges, and these are similar to those that the other countries are facing right now. In some years, there has been a positive trend with solutions identified and implemented; however, in other years, stagnation has occurred. In Kazakhstan, the HTA function is split between two departments of the Ministry of Health, the Department of Science and the Department of Organization of Medical Health. These departments share responsibilities and tasks. A legal framework for HTA was established in 2018, and this should be acknowledged as a milestone, a fundamental element around which a proper HTA framework can be built. This step shows some maturity of the system, which is open to accepting HTA as an effective decision-support tool. In relation to challenges, hospital-based HTA has not been able to find the space that it deserves and it is only performed within one public hospital. Another open challenge is the time for reimbursement decisions, which often at the national level requires too much time. This creates a mismatch with the assessments and investments done at the local level in relation to the implementation of innovative technologies.

Greece is among the last countries to move forward with implementing HTA processes more comprehensively with medicines in 2018 and medical devices in 2022, and they are now moving forward with reforms to align themselves with the new EU regulation on HTA (HTAR). Key challenges are now being mapped through a new project titled *“Strengthening of the National Health Technology Assessment (HTA) Framework in Greece”* led by WHO Europe in collaboration with the European Commission’s Directorate-General for Structural Reform Support (DG REFORM) via the Technical Support Instrument and the Greek Ministry of Health. This project over a period of 18 months will strengthen Greece’s current HTA system for implementation of the HTAR and build capacity in the country to ensure equitable and timely access to cost-effective health technologies that will improve health outcomes and enhance the overall well-being of patients and their families in the country.

In Hungary, capacity building is considered key in the country, and the workforce has to be constantly considered to avoid situations where a specific team or department finds itself understaffed. This is particularly true in the field of HTA as people with the right set of competencies are difficult to retain and having educational programs offered by local academic institutions is helping to mitigate this issue. Countries like Hungary are looking forward to the implementation of the EU HTA Regulation. The joint assessments may reinforce the message about the function of HTA, as well as provide access to unbiased, robust summaries of clinical evidence.

In Poland, the establishment of a legal structure was necessitated by the country joining the EU in 2004 resulting in a need to conform its laws to European legislation. Back then, the country was facing issues related to transparency, objective criteria for pricing and reimbursement of pharmaceuticals, and timelines for reimbursement, especially for innovative therapies. Another considerable facilitator was receiving the Transition Facility Funds for new EU member states. Within a 2-year framework, this financial support was mainly used for capacity building and development of the legal framework, in particular for the new legislation framework for pharmaceuticals. However, in parallel, the Agency for Health Technology Assessment and Tariff System (AOTMiT) was quite involved at an international level with HTAi, the International Network of Agencies for HTA (INAHTA), and the European Network for Health Technology Assessment (EUnetHTA), which gave to the agency national and international relevance. Funding of the agency was secured from the National Health Fund with 0.03 percent of the health insurance budget and its stability was ensured by legally linking the functions of the agency to the basic benefit package. Today, a structured framework to involve stakeholders, especially from public hospitals, is still developing, creating a gap in informed decision-making and slowing the implementation of patient-centered innovations (19).

In Slovenia, HTA is not institutionalized yet and is, like in other countries, behind in the process; there is a working group under the Ministry of Health tasked to achieve this goal as the EU HTA Regulation is facilitating countries to be prepared in that sense. It was highlighted that, in contexts like this, the message that HTA is bringing should be clear as policymakers at higher levels may be not aware of the advantages and benefits that are linked to HTA and may ignore its fundamental importance. This could support the growth of a strong political will, which may ignite the whole process.

In Serbia, the biggest need and challenge relate to the improvement of transparency of decisional processes. HTA could shed some light on these processes, and make them more clear, transparent and evidence-based. The situation was worsened during the COVID-19 pandemic, as decisions were made in reaction to an emergency situation, without any control on the expenditure or proper experts’ consultation.

In Montenegro, HTA is far from being implemented or institutionalized. Issues on transparency of the decisional processes are present, proper competencies and skills in the field of HTA are entirely missing and doubts on the appropriateness of investments for healthcare technology are continuously raised. Also, the lack of accountability makes the context a fertile soil for the current situation to persist.

In Ukraine, in the challenging circumstances of wartime, there was a significant shortage of economic and labor resources in the country, leading to a widening gap between the needs and opportunities for developing the healthcare system. Key challenges include the increase of expertise and capacity, involving young professionals in HTA, establishing a cohesive team to institutionalize the HTA function, and creating an independent HTA agency in the future. Expanding HTA to cover nonpharmaceutical technologies is one of the current priorities, requiring additional training and involvement of stakeholders. To make informed decisions about financing technologies, it is crucial to improve the collection of statistical data on disease incidence and prevalence for various conditions. Developing a network of HTA specialists and introducing new educational programs through Ukrainian higher education institutions is essential for the development of Ukrainian HTA. Additionally, increasing the interest of clinicians and a wider range of stakeholders in the HTA process is vital in line with modern principles of public management in healthcare.

In Slovakia, successful integration of in-depth HTA into the decision-making processes (primarily) for medicinal products was achieved in 2021. With a team of about 30 persons and a budget of about 2 million Euro, the National Institute for Value and Technologies in Healthcare (NIHO) produces around 60 assessments of medicinal products a year following the full EUnetHTA Core Model® 3.0 format (specifically focusing on added benefit assessment and cost-effectiveness assessment). These assessments serve as the foundation for managed entry agreements and decision-making of the appraisal committee for medicinal products. Political support, and an independent source of funding established by law, as well as highly ambitious capacity building, were key to the success. However, the key challenge for Slovak HTA is to maintain this capacity as changes in the political landscape have recently impacted the NIHO´s independence and thus on sustainability of the HTA process.

### How can HTAi support unlocking the opportunities?

Moving from challenges to opportunities, participants were invited to share their views on practical actions or strategies that could be useful to develop and implement to support the advancement of HTA in the region.

The fundamental importance of networking strongly emerged in relation to the opportunity to benefit from international exchanges and also the necessity of involving all stakeholders in the navigating of any journey that HTA undertakes within a country. Being a global association, HTAi has an extended reach and, on the basis of Memorandums of Understanding in place with key partners, such as the WHO, can support local initiatives. This was acknowledged as a unique benefit that countries gain through working with international partners as those entities do not only rely on internal expertise but also have a global reach in terms of experts that can be recruited and involved to support countries.

It was acknowledged that specific groups, like patient associations, can be very effective in bringing a message to policymakers. The message may be translated or adapted somehow, to be sure that there is a common understanding of what the issue is and what the results are that the system can expect from HTA or any activity within the HTA sphere. However, in some contexts, the presence of patient associations is suboptimal or not existing and this represents a structural weakness of the system. It was highlighted how HTAi can be a catalyst for a multistakeholder dialogue and collaboration. An HTAi Interest Group for Patient and Citizen Involvement in HTA (PCIG) already exists and has been extremely active since its establishment, providing not only a platform for discussion and inclusion but also formal methodologies and tools to support patient involvement in HTA processes worldwide (20).

In addition, the significance of fostering connections among early career professionals in HTA on an international scale was pointed out. Establishing these connections is crucial for ensuring ongoing development and enhancement of skills within the HTA field. HTAi plays a pivotal role in this endeavor through its Early Career Network (ECN). This network is designed to support individuals who are just beginning their journey in HTA, providing them with valuable resources, mentorship, and networking opportunities that can significantly aid in their professional development (21). For instance, the HTAi Newcomers’ Guide is an educational resource repository consisting of an open-access collection that spans from introductory materials to specific methodologies. The collection serves as a practical “toolbox” for understanding HTA fundamentals and complements HTAi’s broader capacity building initiatives. The repository includes links to key external resources to support foundational learning in HTA. Currently, the collection features nine main resources, covering topics like HTA basics, research protocol development, ethical evaluation, hospital-based HTA, stakeholder engagement, and economic evaluation (22). Moreover, the HTAi Educational Offers Database (23) serves as a vital resource for newcomers seeking formal education in HTA. This database compiles various educational programs available globally, allowing individuals to find suitable courses that align with their career aspirations and educational needs. By offering access to comprehensive educational resources and fostering a supportive community, HTAi aims to cultivate a new generation of professionals equipped with the knowledge and competencies necessary to contribute effectively to the evolving landscape of HTA.

## Limitations

The policy dialogue series represents the effort of HTAi to further support the development of HTA in specific regions of the world. EECA was targeted given that structural initiatives to connect the countries around HTA are not currently in place or have been discontinued for various reasons. The meeting was able to attract and involve several participants from different countries (Azerbaijan, Greece, Hungary, Kazakhstan, Kyrgyzstan, Montenegro, North Macedonia, Poland, Serbia, Slovakia, Slovenia, Turkey, and Ukraine); however, it needs to be acknowledged that some countries were not represented at all such as Armenia, Albania, Bosnia and Herzegovina, Georgia, Republic of Moldova, and Romania (the invitation was declined as invited people were not able to join either in person or virtual or no response to the invitation was received). According to the meeting format, participants were asked to express their opinions regardless of the official position of the institutions of their countries. This created a free environment for open discussion, but it also represents a disconnect between the individuals and their institutional role. However, the aim of the meeting was not to take official positions or decisions.

## Conclusions and future possible steps

Several key messages emerged from the HTAi Policy Dialogue for EECA discussions. A recurring theme was the need for strong political will and stable institutions to support HTA processes, which remains a significant challenge across the EECA region. Developing robust legal frameworks, as seen in some contexts, is essential for securing funding and building sustainable HTA structures. Rapid capacity development and talent retention are critical, especially in countries where HTA is just beginning; proper educational programs and international collaboration can help address this need. The potential of HTA to increase consistency, legitimacy, and transparency in decision-making must be properly communicated and could be a driver in those contexts where these aspects need to be improved. The role of international organizations, such as WHO, HTAi, and INAHTA, is vital in providing support, facilitating knowledge exchange, and fostering collaboration among countries.

The following actions were proposed to advance HTA in the region. It is important to emphasize that, given the complexity of the systems, those actions could and should be implemented in parallel and not sequentially, to advance more rapidly and be able to develop the different elements synergically, learning from failures once they occur:

- Strengthening political commitment: Advocacy efforts should focus on building strong political will and ensuring that HTA is recognized as a priority at the highest levels of government.
- Developing legal and institutional frameworks: Countries should work toward establishing legal frameworks that support HTA integration into healthcare decision-making processes.
- Capacity building initiatives: Countries should invest in education and training programs to build a skilled HTA workforce, with support from international organizations and academic institutions.
- Enhancing collaboration: Continued engagement with international networks, such as WHO, INAHTA, and HTAi, can provide valuable support and expertise to help countries overcome local challenges.
- Engaging stakeholders: Involving all relevant stakeholders, including patients, healthcare providers, and policymakers, in HTA processes, is essential for ensuring that decisions are transparent, inclusive, and evidence-based.

HTAi will play a crucial role in advancing the aforementioned points by continuing to organize policy dialogues in the EECA region, fostering an environment for open communication and collaboration among stakeholders. These dialogues will serve as a platform for sharing best practices, addressing challenges, and building consensus on the importance of HTA in healthcare decision-making. Additionally, HTAi will support capacity building initiatives by providing access to the HTAi services, programs, and resources designed to enhance the competencies of the HTA workforce.
